# Rapid Global Calibration Technology for Hybrid Visual Inspection System

**DOI:** 10.3390/s17061440

**Published:** 2017-06-19

**Authors:** Tao Liu, Shibin Yin, Yin Guo, Jigui Zhu

**Affiliations:** State Key Laboratory of Precision Measuring Technology and Instruments, Tianjin University, Tianjin 300072, China; liutao1943@tju.edu.cn (T.L.); yin_guo@tju.edu.cn (Y.G.); jiguizhu@tju.edu.cn (J.Z.)

**Keywords:** global calibration, hybrid visual inspection, industrial robot, rapid, homography

## Abstract

Vision-based methods for product quality inspection are playing an increasingly important role in modern industries for their good performance and high efficiency. A hybrid visual inspection system, which consists of an industrial robot with a flexible sensor and several stationary sensors, has been widely applied in mass production, especially in automobile manufacturing. In this paper, a rapid global calibration method for the hybrid visual inspection system is proposed. Global calibration of a flexible sensor is performed first based on the robot kinematic. Then, with the aid of the calibrated flexible sensor, stationary sensors are calibrated globally one by one based on homography. Only a standard sphere and an auxiliary target with a 2D planar pattern are applied during the system global calibration, and the calibration process can be easily re-performed during the system’s periodical maintenance. An error compensation method is proposed for the hybrid inspection system, and the final accuracy of the hybrid system is evaluated with the deviation and correlation coefficient between the measured results of the hybrid system and Coordinate Measuring Machine (CMM). An accuracy verification experiment shows that deviation of over 95% of featured points are less than ±0.3 mm, and the correlation coefficients of over 85% of points are larger than 0.7.

## 1. Introduction

Product quality inspection is playing an increasingly important role in modern industries, including automobile, shipbuilding, aviation, and aerospace industries. The integration of three-dimensional (3D) measurement in industrial development has done a great favor to monitor the overall quality of production processes, shortening the development periods, lowering the product cost, and considerably increasing performance, all of which is greatly helpful in the optimization of the entire manufacturing process. The coordinate measuring machine (CMM), which has the advantage of its precision and flexibility with respect to various measured objects, has become an indispensable part of manufacturing, and is more often applied to electron, automobile, and spaceflight manufacturing, etc. However, CMMs generally work under an environment of constant temperature, constant humidity, dustless conditions, and they require skillful operators. The measuring efficiency of CMMs is also limited by its contact measurement principle. With the rapid progress of the optical image device and the continuous decrease of its cost, the method for inspecting the product quality with the vision-based system is becoming available. Various vision systems have been applied in modern industries, including monocular vision [1], binocular stereo vision [2], structural-light vision [3], and digital projection profilometry [4], etc. And the rocketing development of computer software, hardware and perfection of the computer vision theory have also promoted the performance and efficiency of these vision systems. 

For mass production such as automobile manufacturing, there are some exceptional requirements for the vision inspection systems, such as that the inspection process should be performed in-line, and that the large sum of critical dimensions to be measured are distributed all over the car body, including the underbody. Industrial robots, featured as an economical and flexible orienting device, offer a new idea to solve these problems. The robotic visual inspection system is not only economically promising, but also combines the industrial robot’s high-flexibility and the visual sensor’s high-throughput and relatively high accuracy. However, for the dimensions that are beyond the robot’s reachable volume, such as the featured points under the car body, some dedicated vision sensors are added. Also, for the inspection system, the coordinate system used to define the car body is determined by measuring some locating holes, which requires a higher measuring accuracy and is also realized by adding dedicated sensors. As these sensors are usually fixed on a steel structure, we name them stationary sensors in this paper, while the sensors mounted on the robot flange are named flexible sensors. Hybrid visual inspection systems, which consist of flexible sensors and stationary sensors, have been commonly used in mass production. 

For the car body-in-white in-line hybrid inspection system, the robot orients the flexible sensor to measure featured points on the car body surface, such as side panel, roof, front and rear windshield, while the stationary sensors measure the featured points on the underbody and the locating holes. For a typical hybrid visual inspection system as described above, there are two kinds of vision sensors and an industrial robot, all of which have their defined reference coordinates in the system. The final measured results of the hybrid system are usually unified and analyzed in a global coordinate system, which is defined with several locating points on the product. So, the global calibration to establish the relationship between different coordinate systems is of great significance for the final measuring accuracy of the hybrid inspection system. Moreover, the hybrid visual inspection system is usually integrated in the industrial field, and the global calibration should be performed in the workshop, which brings forward higher requirement to the efficiency and robustness of the calibration method. Also, vision sensors may be uninstalled and replaced during the system’s periodical maintenance, and a rapid global calibration method is an indispensable function for the hybrid inspection system.

In order to realize the global calibration of the measurement system with several visual sensors, Kitahara et al. [5] combined a calibration board and a 3D laser-surveying instrument to realize camera calibration in a large-scale space. Lu and Li [6] utilized a theodolite coordinate measurement system to realize the global calibration. Liu and Lin [7] employed a laser tracker (FARO, Orlando, FL, USA) and a special 3D target to conduct the global calibration. Still, all the above methods require an additional high precision 3D measurement apparatus to assist in the calibration, and although they can acquire relatively high calibration accuracy, they are quite complicate, high-cost and not very effective. And when the stationary sensors are installed on a complex fixture, the light path of these apparatuses may be obstructed easily. Moreover, sensors may only be uninstalled and replaced during the system’s periodical maintenance and calibration with help of sophisticated auxiliary apparatuses that are inconvenient and time-consuming. To solve this problem, some other researchers have been searching for calibration approaches without involving sophisticated apparatus. Liu et al. [8] computed the coordinate transformation from each vision sensor to a global coordinate frame according to the co-linearity of the laser spots at each spot laser position. Xie and Wei [9] unified the coordinate frames of the two cameras by a flexible target composed of two short 1D bars. The above two methods use a simple and flexible apparatus for global calibration, which are not only convenient and fast but also have a relatively high accuracy. However, these methods mainly discussed the calibration method of the transformation relationship among several visual sensors or cameras, and the measured results are unified in the coordinate system of a sensor, not a global reference frame. Furthermore, there is no flexible sensor applied in the systems discussed.

In this paper, we proposed a rapid global calibration method for hybrid visual inspection systems. System construction and measurement principle of the hybrid visual inspection system are presented first. The global calibration of the hybrid system is divided into two steps, calibrating the flexible sensor first, and then calibrating stationary sensors. Based on the robot kinematic principle, a flexible sensor global calibration method is proposed, which is performed with the aid of a standard sphere. Then, with the calibrated flexible sensor, the stationary sensors are calibrated one by one based on the homography principle. An auxiliary target with a 2D planar pattern is used as the calibration target, and the global calibration of a stationary sensor can be finished with just one robot movement and two images captured by the flexible and stationary sensor, which is very simple to operate and high-efficient. No sophisticated equipment is adopted during the global calibration process, and it is convenient to re-perform during the hybrid system periodical maintenance. An accuracy verification experiment is performed and an error compensation method is proposed for the hybrid inspection system, and the final accuracy of the hybrid system is evaluated with the deviation and correlation coefficient between the measured results of the hybrid visual inspection system and the CMM.

The remainder of this paper is organized as follows: Section 2 is a brief introduction to the system construction and measurement principle of the hybrid visual inspection system. A detailed description of the system rapid global calibration method is given in Section 3, including flexible and stationary sensor global calibration. In Section 4, the accuracy verification experiment is carried out, and an error compensation method is proposed for the hybrid inspection system. The conclusions are given in Section 5.

## 2. Principle of the Hybrid Visual Inspection System

### 2.1. System Principle

**Figure 1:**
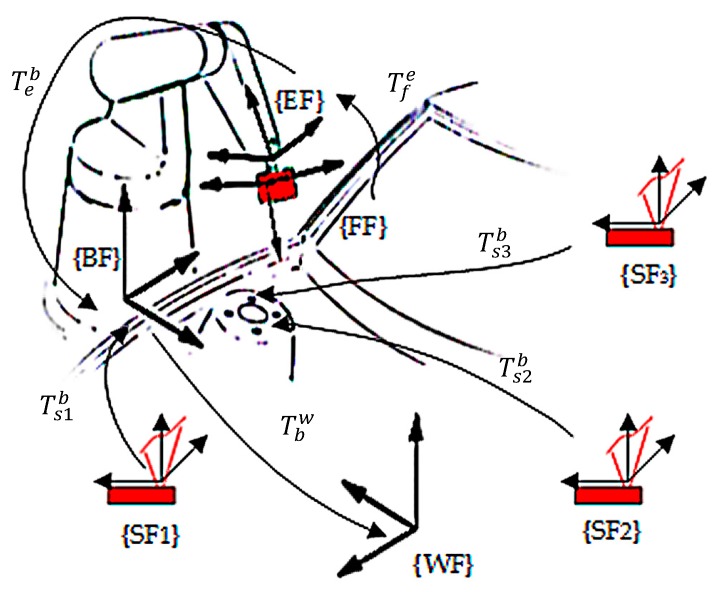
Schematic of the hybrid visual inspection system.

As shown in Figure 1, a typical hybrid visual inspection system mainly consists of a six degrees of freedom industrial robot, a flexible vision sensor, and several stationary vision sensors. The flexible sensor is oriented by the industrial robot to measure featured points on the workpiece surface, and the stationary sensors are responsible for measuring the featured points on the underbody or the points beyond the robot’s reachable volume. Also, as the measured results are usually unified and accessed in the workpiece coordinate system, which is established with several locating points on it, these locating points are measured with the stationary sensors in order to guarantee a good accuracy.

Line-structured laser sensors are used in the hybrid visual inspection system discussed in this paper. The main measuring principle of a line-structured laser sensor is the optical triangulation principle [10]. The shape of the object to be measured can be modulated by a laser projector from one direction and captured by the camera from another. The 3D geometrical dimensions of the laser stripe can be calculated directly.

As shown in Figure 1, the coordinate systems of the hybrid visual inspection system consist of a workpiece frame (WF), robot base frame (BF), end-effector frame (EF), flexible sensor frame (FF), and stationary sensor frames (SFi). For the inspection system described in this paper, BF is a fixed frame and is regarded as the global reference frame; establishing the transform relationships between BF and the sensor frames is the global calibration process described in this paper; and the measured results of the flexible and stationary sensors are unified in BF first. WF is constructed separately on each workpiece to be measured; when the workpiece is fixed and clamped in the inspection station, several stationary sensors measure the locating points on it, then the transformation between BF and WF ($T_{b}^{w}$) is determined with the construction method described in Section 2.2. 

For a featured point measured by the flexible sensor, the mapping relationship between coordinate $P_{w1}$ in WF and $P_{f}$ in FF is expressed as follows:

(1) $$P_{w1}=T_{b}^{w}\cdot T_{e}^{b}\cdot T_{f}^{e}\cdot P_{f}$$

where $T_{f}^{e}$ is the transformation between FF and EF, which is also called hand-eye relationship; $T_{e}^{b}$ is the transformation between EF and BF, which could be obtained from robot angular values and the forward kinematic model.

For a featured point measured by a stationary sensor, the mapping relationship between coordinate $P_{w2}$ in WF and $P_{Si}$ in SFi is expressed as follows:

(2) $$P_{w2}=T_{b}^{w}\cdot T_{si}^{b}\cdot P_{si}$$

where $T_{si}^{b}$ is the transformation between SFi and BF. An individual SFi is defined on each stationary sensor and $T_{si}^{b}$ is to be determined in the system global calibration process.

Before such a hybrid visual inspection system being applied in the industrial measurement, the following three calibration procedures should be performed. First, the intrinsic and extrinsic parameters calibration of the line-structured laser sensor should be calibrated [11,12]. Second, it is necessary to determine the transformation between the flexible sensor and robot end-flange. Third, the relationship between each stationary sensor and the robot base frame should be obtained. The last two procedures can be regarded as the global calibration of the laser sensors, with which the measured results of the laser sensors can be unified to the global frame (robot base frame). As the sensor’s intrinsic and extrinsic parameters in this hybrid system have been pre-calibrated, only the global calibration is needed to be performed in the industrial field. Also, when a vision sensor is uninstalled and replaced with another sensor during the system maintenance, the global calibration of this sensor should be re-performed.

### 2.2. Reference Point System (RPS) and Workpiece Frame Definition

As mentioned before, the measured results of the hybrid visual system are generally analyzed in WF. In this paper, WF is defined based on the datum features on the workpiece itself. In an industry, especially in the automobile industry, RPS is frequently used for the measurement systems to ensure a comparable result worldwide. For a complete vehicle, reference points are defined on the workpiece, and these points allow for an alignment in 3D space. Once the object is aligned to the theoretical coordinate system of the model (AutoCad data), one is able to compare any desired point and detect manufacturing errors on the product.

**Figure 2:**
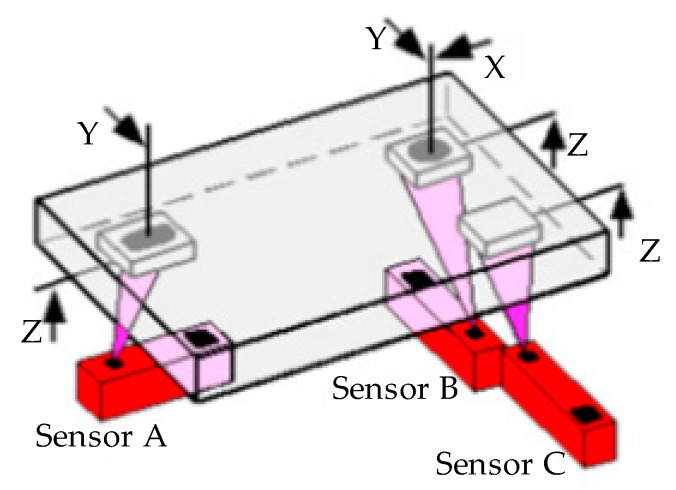
The 3-2-1 principle used to define workpiece frame (WF).

Every rigid body possesses six degrees of freedom in three-dimensional space: three translational degrees of freedom parallel to the axes of a reference system and three rotatory degrees of freedom around the axes. In order to support a non-rotationally-symmetric body in a uniquely determinate manner, it must be fixed in all six possible directions of movement. The 3-2-1-rule provides for such unique fixing. As shown in Figure 2, the 3-2-1 locating rule is used to determine the location of the workpiece in 3D space, and only three stationary sensors are necessary to determine the position of the part: Sensor A measures a slot; Sensor B measures a hole; Sensor C measures a point on a plane. This completes the 3-2-1 locating as follows:

(3) locating Points in Z axis (plane) (measures Z translation and rotation about Y and X)
  Sensor A—slot center Z value
  Sensor B—hole center Z value
  Sensor C—range Z value
(2) locating Points in Y axis (line) (measures Y translation and rotation about Z)
  Sensor A—slot center Y value
  Sensor B—hole center Y value
(1) locating Point in X axis (point) (measures X translation)
  Sensor B—hole center X value

Coordinate of the datum features $P_{WA}$ ($x_{WA}$, $y_{WA}$, $z_{WA}$), $P_{WB}$ ($x_{WB}$, $y_{WB}$, $z_{WB}$), $P_{WC}$ ($x_{WC}$, $y_{WC}$, $z_{WC}$) in WF can be obtained from the CAD model. When determining WF, the measured results of the three stationary sensors are unified in BF first, and the coordinates of the datum features in BF are $P_{BA}$ ($x_{BA}$, $y_{BA}$, $z_{BA}$), $P_{BB}$ ($x_{BB}$, $y_{BB}$, $z_{BB}$), $P_{BC}$ ($x_{BC}$, $y_{BC}$, $z_{BC}$). When the transformation from WF to BF is $T_{b}^{w}=\left[\begin{array}{cc} R & T \end{array}\right]=\left[\begin{array}{cccc} r_{11} & r_{12} & r_{13} & t_{1}\\ r_{21} & r_{22} & r_{23} & t_{2}\\ r_{31} & r_{32} & r_{33} & t_{3} \end{array}\right]$, with the measured datum features, we have:

(3) $$\{\begin{array}{l} F_{1}=r_{21}\cdot x_{BA}+r_{22}\cdot y_{BA}+r_{23}\cdot z_{BA}+t_{2}-y_{WA}=0\\ F_{2}=r_{31}\cdot x_{BA}+r_{32}\cdot y_{BA}+r_{33}\cdot z_{BA}+t_{3}-z_{WA}=0\\ F_{3}=r_{11}\cdot x_{BB}+r_{12}\cdot y_{BB}+r_{13}\cdot z_{BB}+t_{1}-x_{WB}=0\\ F_{4}=r_{21}\cdot x_{BB}+r_{22}\cdot y_{BB}+r_{23}\cdot z_{BB}+t_{2}-y_{WB}=0\\ F_{5}=r_{31}\cdot x_{BB}+r_{32}\cdot y_{BB}+r_{33}\cdot z_{BB}+t_{3}-z_{WB}=0\\ F_{6}=r_{31}\cdot x_{BC}+r_{32}\cdot y_{BC}+r_{33}\cdot z_{BC}+t_{3}-z_{WC}=0 \end{array}$$

The rotation matrix *R* satisfies the orthogonal constraint condition described as follows:

(4) $$\{\begin{array}{c} f_{1}=r_{11}\cdot r_{11}+r_{21}\cdot r_{21}+r_{31}\cdot r_{31}-1=0\\ f_{2}=r_{12}\cdot r_{12}+r_{22}\cdot r_{22}+r_{32}\cdot r_{32}-1=0\\ f_{3}=r_{13}\cdot r_{13}+r_{23}\cdot r_{23}+r_{33}\cdot r_{33}-1=0\\ f_{4}=r_{11}\cdot r_{21}+r_{12}\cdot r_{22}+r_{13}\cdot r_{23}=0\\ f_{5}=r_{11}\cdot r_{31}+r_{12}\cdot r_{32}+r_{13}\cdot r_{33}=0\\ f_{6}=r_{21}\cdot r_{31}+r_{22}\cdot r_{32}+r_{23}\cdot r_{33}=0 \end{array}$$

From Equations (3) and (4), $\left[\begin{array}{cc} R & T \end{array}\right]$ can be obtained by minimizing the following function:

(5) $$E=\sum_{i=1}^{6}\left(F_{i}^{2}+Mf_{i}^{2}\right)$$

where *M* is the penalty factor. Minimizing Equation (5) is a nonlinear minimization problem, which can be solved with the Levenberg–Marquardt algorithm [13,14,15].

## 3. Rapid Global Calibration Method

Global calibration of the laser sensors (including flexible sensors and stationary sensors) are generally finished in the industrial field, and may need to be re-performed during the system periodical maintenance, so a streamlined and efficient global calibration method is necessary for the hybrid visual inspection system. Also, with the on-site industrial environment being more and more complicated, calibration with a sophisticated apparatus such as a laser tracker or an articulated arm CMM is not applicable; it is thus preferred that the hybrid system can be self-calibrated with the aid of simple apparatuses. In this paper, we propose a rapid global calibration technology for the hybrid visual inspection system. Global calibration of the flexible sensor is finished by using a standard sphere as the calibration target. Then, with the calibrated flexible sensor and an auxiliary planar target, the stationary sensors are calibrated based on the homography principle.

### 3.1. Flexible Sensor Global Calibration

In this method, the global calibration of the flexible sensor is to determine the rotation matrix $R_{F}$ and translation vector $T_{F}$ between the flexible sensor frame and robot end-flange frame, which is also called hand-eye calibration. A standard sphere is used as a reference through measuring the fixed sphere-center at different robot poses by the flexible sensor. $R_{F}$ and $T_{F}$ can be solved through the constraint of a fixed space point [16].

For the standard sphere fixed in the robot working space, the relationship between the coordinate of the sphere-center $X_{B}={\left[\begin{array}{ccc} x_{B} & y_{B} & z_{B} \end{array}\right]}^{T}$ in the robot base frame and $X_{F}={\left[\begin{array}{ccc} x_{F} & y_{F} & z_{F} \end{array}\right]}^{T}$ in the flexible laser sensor frame is as follows:

(6) $$\left[\begin{array}{c} X_{B}\\ 1 \end{array}\right]=\left[\begin{array}{cc} R_{0} & T_{0}\\ 0 & 1 \end{array}\right]\cdot \left[\begin{array}{cc} R_{F} & T_{F}\\ 0 & 1 \end{array}\right]\cdot \left[\begin{array}{c} X_{F}\\ 1 \end{array}\right]$$

where $R_{0}$ and $T_{0}$ are the rotation matrix and translation vector from robot end-flange to robot base. $R_{F}$ and $T_{F}$ are the rotation matrix and translation vector from the flexible sensor to end-flange. Equation (6) can be expanded as:

(7) $$X_{B}=R_{0}\cdot R_{F}\cdot X_{F}+R_{0}\cdot T_{F}+T_{0}$$

Controlling the robot and making the flexible sensor measure the sphere-center twice, we can obtain the following equations:

(8) $$\{\begin{array}{l} X_{B}=R_{01}\cdot R_{F}\cdot X_{F1}+R_{01}\cdot T_{F}+T_{01}\\ X_{B}=R_{02}\cdot R_{F}\cdot X_{F2}+R_{02}\cdot T_{F}+T_{02} \end{array}$$

If the robot pose remains unchanged during these two measurements (motion with only translation), that is $R_{01}=R_{02}=R_{0}$, by subtracting the two equations in Equation (8) we can get:

(9) $$R_{0}\cdot R_{F}\cdot (X_{F1}-X_{F2})+T_{01}-T_{02}=0$$

By repeating the measurements by several robot translation movements, we can collect several sets of experimental data and obtain the following equation:

(10) $$R_{F}\cdot \left[\begin{array}{cccc} X_{F1}-X_{F2} & X_{F1}-X_{F3} & \cdots & X_{F1}-X_{Fn} \end{array}\right]=R_{0}^{T}\cdot \left[\begin{array}{cccc} T_{02}-T_{01} & T_{03}-T_{01} & \cdots & T_{0n}-T_{01} \end{array}\right]$$

Let $P_{1}=\left[\begin{array}{cccc} X_{F1}-X_{F2} & X_{F1}-X_{F3} & \cdots & X_{F1}-X_{Fn} \end{array}\right]$, $P_{0}=R_{0}^{T}\cdot \left[\begin{array}{cccc} T_{02}-T_{01} & T_{03}-T_{01} & \cdots & T_{0n}-T_{01} \end{array}\right]$, and Equation (10) can be rewritten as $R_{F}P_{1}=P_{0}$. According to the Singular Value Decomposition (SVD) method, the following equation should be established:

(11) $$K=d_{0}\cdot d_{1}^{T}=(P_{0}-C_{0})\cdot {\left(P_{1}-C_{1}\right)}^{T}$$

where $C_{0}=\frac{\sum_{i=1}^{n}P_{0}}{n}$ and $C_{1}=\frac{\sum_{i=1}^{n}P_{1}}{n}$. The K can be decomposed into $K=U\cdot \Lambda \cdot V^{T}$, and the solution of matrix $R_{F}$ will be set to $R_{F}=U\cdot V^{T}$.

After determining $R_{F}$, we can control the robot and make the flexible sensor measure the same fixed point from different robot poses, and obtain the following equation from Equation (8):

(12) $$(R_{02}-R_{01})\cdot T_{F}=R_{01}\cdot R_{F}\cdot X_{F1}-R_{02}\cdot R_{F}\cdot X_{F2}+T_{01}-T_{02}$$

By repeating the robot pose-changing movement several times and gathering several sets of experimental data, a linear equation is obtained in the form of matrix as:

(13) $$\left[\begin{array}{c} R_{02}-R_{01}\\ R_{03}-R_{01}\\ \cdots \\ R_{0n}-R_{01} \end{array}\right]\cdot T_{F}=\left[\begin{array}{c} R_{01}\cdot R_{F}\cdot X_{F1}-R_{02}\cdot R_{F}\cdot X_{F2}+T_{01}-T_{02}\\ R_{01}\cdot R_{F}\cdot X_{F1}-R_{03}\cdot R_{F}\cdot X_{F2}+T_{01}-T_{03}\\ \cdots \\ R_{01}\cdot R_{F}\cdot X_{F1}-R_{0n}\cdot R_{F}\cdot X_{F2}+T_{01}-T_{0n} \end{array}\right]$$

Equation (13) is a system of linear equations in the form of $AT_{F}=B$. As long as the coefficient matrix in Equation (13) is nonsingular, $T_{F}$ could be solved by means of the Least-squares method as follows:

(14) $$T_{F}={\left(A^{T}\cdot A\right)}^{-1}A^{T}\cdot B$$

Till now, rotation matrix $R_{F}$ and translation vector $T_{F}$ from the flexible sensor to end-flange have been determined, and the global calibration of the flexible sensor has been finished.

The method for measuring the sphere-center is described in Reference [17], in which the sphere radius should be known in order to obtain the sphere-center at one robot position by one laser shoot. In order to guarantee the measuring accuracy, the laser should project on *R*/4 of the sphere surface.

### 3.2. Stationary Sensors Global Calibration

After the global calibration of the flexible sensor, the stationary sensors’ global calibration can be carried out with the aid of an auxiliary target and the calibrated flexible sensor. Global calibration of the stationary sensors is based on the camera pinhole model and homography principle, which is described in this section first.

#### 3.2.1. Camera Pinhole Model and Homography

For the vision sensors in the hybrid visual inspection system, the usual pinhole is used to model the cameras in the visual sensors. The relationship between the coordinate of a spatial point $P_{s}$ and its corresponding image point $P_{c}$ can be given as follows:

(15) $$sP_{c}=A_{c}T_{c}P_{s}$$

where *s* is an arbitrary scale factor, $P_{s}={\left[\begin{array}{cc} x_{s} & \begin{array}{cc} y_{s} & \begin{array}{cc} z_{s} & 1 \end{array} \end{array} \end{array}\right]}^{T}$ is the homogeneous coordinate of a spatial point in the object space, and $P_{c}={\left[\begin{array}{ccc} u_{c} & u_{c} & 1 \end{array}\right]}^{T}$ is the corresponding homogeneous coordinate in the camera image space. $T_{c}=\left[\begin{array}{cccc} r_{c11} & r_{c12} & r_{c13} & t_{cx}\\ r_{c21} & r_{c22} & r_{c23} & t_{cy}\\ r_{c31} & r_{c32} & r_{c33} & t_{cz}\\ 0 & 0 & 0 & 1 \end{array}\right]$ is the camera extrinsic matrix, which represents the transformation from the object coordinate system to the camera coordinate system, and $A_{c}=\left[\begin{array}{cccc} f_{cx} & \gamma_{c} & u_{c0} & 0\\ 0 & f_{cy} & v_{c0} & 0\\ 0 & 0 & 1 & 0 \end{array}\right]$ is the camera intrinsic matrix which represents the transformation from the camera coordinate system to the image space, with the focal length in pixels ($f_{cx}$, $f_{cy}$), the principal point ($u_{c0}$, $v_{c0}$), and the skew factor $\gamma_{c}$.

Homography matrix, which is a nonsingular 3 × 3 matrix, is used to define the homogeneous linear transformation from a plane to another in the projective space [18]. In this paper, planar homography is used to express the homogeneous linear relationship between the camera image plane and the target plane. According to the pinhole camera model, coordinates of a target point on the target plane and its image point on the camera image plane satisfy the following relationship:

(16) $$\begin{array}{cc} s\left[\begin{array}{c} u_{c}\\ v_{c}\\ 1 \end{array}\right] & =\left[\begin{array}{cccc} f_{cx} & \gamma_{c} & u_{c0} & 0\\ 0 & f_{cy} & v_{c0} & 0\\ 0 & 0 & 1 & 0 \end{array}\right]\left[\begin{array}{cccc} r_{c11} & r_{c12} & r_{c13} & t_{cx}\\ r_{c21} & r_{c22} & r_{c23} & t_{cy}\\ r_{c31} & r_{c32} & r_{c33} & t_{cz}\\ 0 & 0 & 0 & 1 \end{array}\right]\left[\begin{array}{c} x_{t}\\ y_{t}\\ 0\\ 1 \end{array}\right]\\ & =\left[\begin{array}{ccc} f_{cx} & \gamma_{c} & u_{c0}\\ 0 & f_{cy} & v_{c0}\\ 0 & 0 & 1 \end{array}\right]\left[\begin{array}{ccc} r_{c11} & r_{c12} & t_{cx}\\ r_{c21} & r_{c22} & t_{cy}\\ r_{c31} & r_{c32} & t_{cz} \end{array}\right]\left[\begin{array}{c} x_{t}\\ y_{t}\\ 1 \end{array}\right] \end{array}$$

$H_{t}^{c}$ stands for the homography matrix from the target plane to the camera image plane:

(17) $$H_{t}^{c}=A[\begin{array}{ccc} r_{c1} & r_{c2} & t_{c} \end{array}]=\left[\begin{array}{ccc} f_{cx} & \gamma_{c} & u_{c0}\\ 0 & f_{cy} & v_{c0}\\ 0 & 0 & 1 \end{array}\right]\left[\begin{array}{ccc} r_{c11} & r_{c12} & t_{cx}\\ r_{c21} & r_{c22} & t_{cy}\\ r_{c31} & r_{c32} & t_{cz} \end{array}\right]$$

Thus, Equation (16) can be rewritten as:

(18) $$sP_{c}=H_{t}^{c}P_{t}$$

where $P_{t}={\left[\begin{array}{ccc} x_{t} & y_{t} & 1 \end{array}\right]}^{T}$ is the coordinate of a point on the target plane and $P_{c}={\left[\begin{array}{ccc} u_{c} & v_{c} & 1 \end{array}\right]}^{T}$ is the corresponding camera image coordinate.

When $H_{t}^{c}=\left[\begin{array}{ccc} h_{1} & h_{2} & h_{3} \end{array}\right]=A\left[\begin{array}{ccc} r_{c1} & r_{c2} & t_{c} \end{array}\right]$, and when *A* and $H_{t}^{c}$ have been obtained, the extrinsic matrix (transformation from the target coordinate system to the camera coordinated system) can be calculated with the following method:

(19) $$\{\begin{array}{c} r_{c1}=\gamma A^{-1}h_{1}\\ r_{c2}=\gamma A^{-1}h_{2}\\ r_{c3}=r_{1}\times r_{2}\\ t_{c}=\gamma A^{-1}h_{3} \end{array}$$

As the target is always in front of the camera image, if we get $t_{c}(3)>0$ in Equation (19), then we should let $\gamma =1/\left\|A^{-1}h_{1}\right\|=1/\left\|A^{-1}h_{2}\right\|$. If $t_{c}(3)<0$, and then $\gamma =-1/\left\|A^{-1}h_{1}\right\|=-1/\left\|A^{-1}h_{2}\right\|$, we can calculate the extrinsic matrix again [19].

#### 3.2.2. Principle of Stationary Sensor Global Calibration

**Figure 3:**
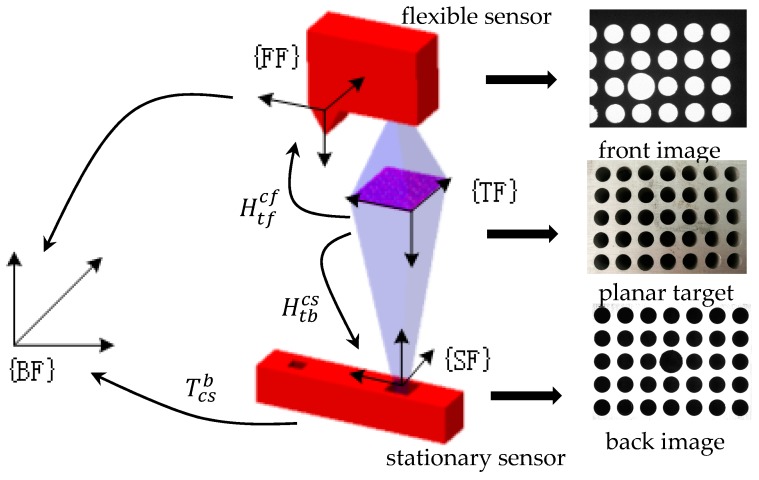
Principle of the stationary sensor global calibration.

The principle of stationary sensor global calibration is illustrated in Figure 3. After the global calibration of the flexible sensor, an auxiliary target with a 2D planar pattern is mounted on the robot end-flange, and the planar pattern is fixed on the working distance of the flexible sensor. The planar target is made of aluminum and has 5 × 7 holes machined on the target; the holes are manufactured with an accuracy of 0.05 mm. As shown in Figure 3, the front image and the back image are captured by the flexible sensor and the stationary sensor, respectively, and a picture of the planar target captured with a camera has also been provided.

When calibrating a stationary sensor, the robot orients the flexible sensor and the calibration target to above of the stationary sensor, where the planar pattern is also on the working distance of the stationary sensor. Then, the flexible sensor captures the image of the front side of the planar pattern, and the stationary sensor captures the image of the back side, and the image coordinates of the hole-centers on the flexible and stationary sensors are extracted by ellipse-fitting methods [20,21]. As the hole arrays are manufactured by precision machining, coordinates of the hole centers on the target coordinate system are known from the CAD model. With the hole-centers’ image coordinates and object coordinates, we can obtain the following equation:

(20) $$\{\begin{array}{c} s_{1}P_{cf}=H_{tf}^{cf}P_{tf}\\ s_{2}P_{cs}=H_{tb}^{cs}P_{tb} \end{array}$$

where $P_{cf}={\left[\begin{array}{ccc} u_{cf} & v_{cf} & 1 \end{array}\right]}^{T}$ and $P_{cs}={\left[\begin{array}{ccc} u_{cs} & v_{cs} & 1 \end{array}\right]}^{T}$ are the image coordinates of the hole array on the flexible and stationary sensors; $P_{tf}={\left[\begin{array}{ccc} x_{tf} & y_{tf} & 1 \end{array}\right]}^{T}$ and $P_{tb}={\left[\begin{array}{ccc} x_{tb} & y_{tb} & 1 \end{array}\right]}^{T}$ are the coordinate of hole centers on the front and back sides of the target plane; $H_{tf}^{cf}$ is the homography matrix from the front side of the target plane to the flexible sensor’s camera image plane; and $H_{tb}^{cs}$ is the homography matrix from the back side of the target plane to the stationary sensor’s camera image plane.

Let $T_{tf}^{cf}=\left[\begin{array}{cccc} r_{f1} & r_{f2} & r_{f3} & t_{f} \end{array}\right]$ be the extinct transformation matrix from the front side of the target to the flexible sensor, $T_{tb}^{cs}=\left[\begin{array}{cccc} r_{s1} & r_{s2} & r_{s3} & t_{s} \end{array}\right]$ be the extinct matrix from the back side of the target to the stationary sensor, and $T_{tb}^{tf}=\left[\begin{array}{cc} \begin{array}{ccc} r_{t1} & r_{t2} & r_{t3} \end{array} & t_{t} \end{array}\right]$ be the transformation from the back side of the target to the front side, which is pre-calibrated with CMM. According to Equation (17), $H_{tf}^{cf}\;$ and $H_{tb}^{cs}$ can be rewritten as:

(21) $$\{\begin{array}{c} H_{tf}^{cf}=A_{cf}\cdot [\begin{array}{ccc} r_{f1} & r_{f2} & t_{f} \end{array}]\\ H_{tb}^{cs}=A_{cs}\cdot [\begin{array}{ccc} r_{s1} & r_{s2} & t_{s} \end{array}] \end{array}$$

As the camera intrinsic matrices of the flexible and stationary sensors have been pre-calibrated, when the homography $H_{tf}^{cf}$ and $H_{tb}^{cs}$ have been determined, we can calculate the extinct transformation matrixes $T_{tf}^{cf}$ and $T_{tb}^{cs}$ according to Equation (19).

**Figure 4:**
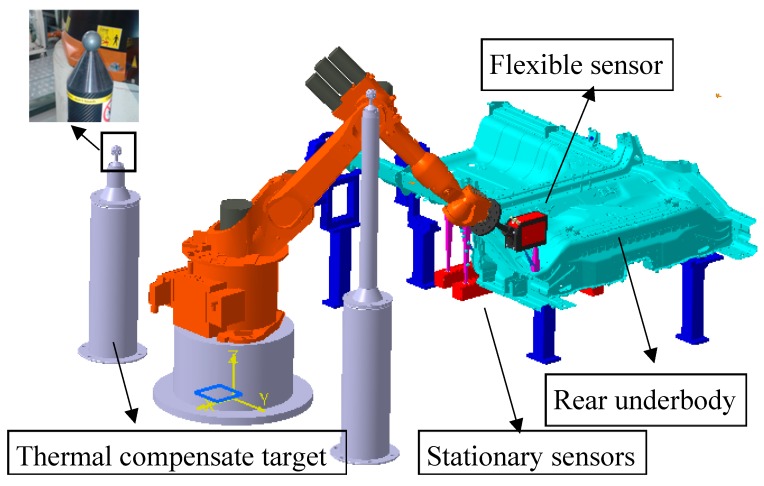
3D model of the hybrid visual inspection station.

As shown in Figure 4, we can obtain the transformation from the target front side to the robot base frame from two different coordinate conversion chains:

(22) $$\{\begin{array}{c} T_{tf}^{b}=T_{e}^{b}\cdot T_{cf}^{e}\cdot T_{tf}^{cf}\\ T_{tf}^{b}=T_{cs}^{b}\cdot T_{tb}^{cs}\cdot T_{tf}^{tb} \end{array}$$

where $T_{e}^{b}$ is the robot end-flange posture with respect to the robot base, which can be obtained from the robot forward kinematics; $T_{cf}^{e}$ is the robot hand-eye relationship, which is calibrated in Section 3.1; and $T_{cs}^{b}$ is the transformation from the stationary sensor to the robot base, which is to be determined during the stationary sensor global calibration.

According to Equation (22), $T_{cs}^{b}$ can be calculated as:

(23) $$T_{cs}^{b}=T_{e}^{b}\cdot T_{cf}^{e}\cdot T_{tf}^{cf}\cdot {\left(T_{tb}^{cs}\cdot T_{tf}^{tb}\right)}^{-1}$$

With the calibrated flexible sensor and an auxiliary target, global calibration of a stationary sensor can be completed with just one robot movement and two images captured by the flexible and stationary sensors. The whole calibration process only needs one person and takes about five minutes to complete, and it is simple to operate as well as highly efficient. The auxiliary target is installed on the connecting rod directly and, once installed, the robot can move the target and flexible sensor to calibrate all of the stationary sensors. The auxiliary target is also convenient to reinstall during the hybrid system’s periodical maintenance.

## 4. Experiment and Analysis

### 4.1. Experiment Setup

As mentioned at the beginning, hybrid visual inspection systems have been commonly used in mass production, especially in automobile manufacturing. In order to verify the effectiveness and efficiency of the global calibration method proposed in this paper, we tested an on-site hybrid visual inspection station for the car body rear underbody panel. The car body rear underbody panel is about 1500 mm in length and 1200 mm in width. A 3D model of the inspection station is shown in Figure 4, and it is on the last station of a production line for the rear underbody panel. The car body rear underbody panel is assembled and welded on the front stations first, and then brought to the last station for quality inspection. Dimensional information is saved on a computer and can be queried by quality engineers; product that is significantly dimensionally out of tolerance will trigger line-stopping and be removed for repair.

This hybrid visual inspection system consists of a flexible sensor mounted on a KUKA KR16-2 industrial robot (KUKA, Augsburg, Germany), and 10 stationary sensors fixed on the baseboard of the fixture. According to the specifications, the typical repeatability of this robot is about ±0.06 mm. The robot uses the flexible sensor to measure 48 points around the rear underbody panel, and the stationary sensors measure the featured points under the panel, which cannot be reached by the robot. Also, three stationary sensors were adopted to measure the locating points under the panel, and the workpiece frame was established on each panel with the principle presented in Section 2.2.

### 4.2. Calibration Results

Before carrying out the experimental test, the global calibration of the flexible and stationary sensors were performed first. As shown in Figure 4, there were two standard spheres fixed in the robot workspace. The standard sphere is extremely hard with a low coefficient of thermal expansion, and is designed for real-time thermal error compensation of the industrial robot. Also, it can be used as the reference point in the global calibration of the flexible sensor.

First, the industrial robot used the flexible sensor to scan the standard sphere in four different positions, and the pose of the end-flange remained unchanged during these four motions. Then, the robot used the flexible sensor to scan the standard sphere in six other different positions, and the pose of the end-flange changed during these six motions. With the robot end-flange poses read from the robot control system and the positions of the sphere-center measured in the flexible sensor frame, the rotation matrix and translation vector from the flexible sensor to the end-flange was calibrated as:

$$\left[\begin{array}{cccc} 0.647394 & 0.01769 & 0.76195 & -46.037782\\ -0.014428 & 0.999836 & -0.010954 & -4.455687\\ -0.762019 & -0.003901 & 0.647543 & 413.045474\\ 0 & 0 & 0 & 1 \end{array}\right]$$

**Figure 5:**
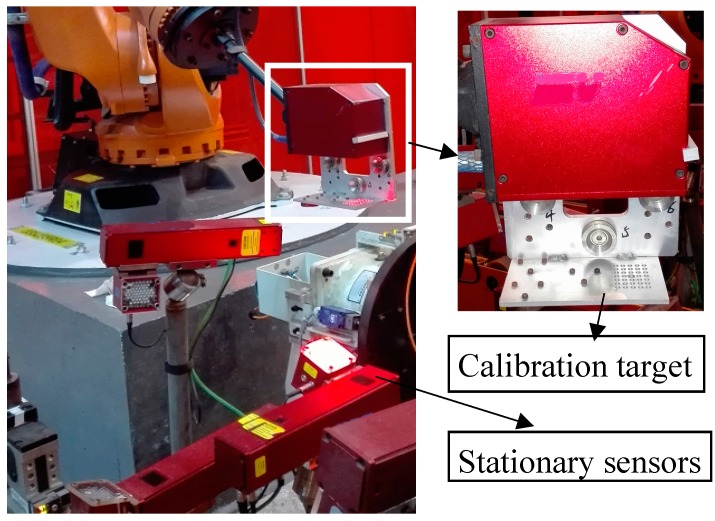
Global calibration of the stationary sensors.

After the flexible sensor was calibrated, an auxiliary target was mounted on the robot connection rod as shown in Figure 5, and the stationary sensors were calibrated with the method presented in Section 3.2, one by one. First, the robot oriented the flexible sensor and the calibration target to above of the stationary sensor, where the planar pattern was also within the working distance of the stationary sensor. Then, the flexible sensor captured the image of the front side of the planar pattern, and the stationary sensor captured the image of the back side. With the captured image, the transformation from the target to the flexible and station sensors was calculated. The robot end-flange pose was also read from the robot control system, and the transformation between the stationary sensor and the robot base was determined with Equation (23).

### 4.3. Accuracy Validation Experiments

**Figure 6:**
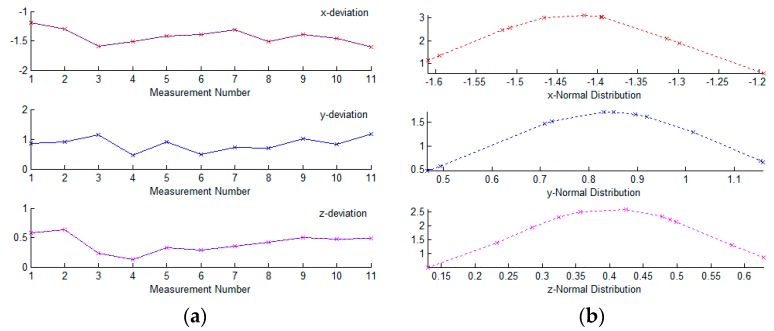
Deviations between measured values of hybrid visual system and Coordinate Measuring Machine (CMM): (**a**) 10 origin datums of a hole; (**b**) The corresponding normal distribution fitting.

With the calibrated hybrid system, the rear underbody panel was measured at the inspection station. As mentioned above, 58 featured points were measured on the panel, including holes, edges, and flanges. Forty-eight points were measured by the flexible sensor and 10 points were measured by the stationary sensors. Once the underbody panel was fixed and clamped on the fixture, three stationary sensors measured the locating points and established the workpiece frame first, then the other seven stationary sensors and the flexible sensor measured the other points, and coordinates of the featured points were unified in the workpiece frame. After being measured at the inspection station, the underbody panel was also measured by CMM. Due to the high accuracy, measured results of CMM were regarded as reference values, and deviations between the measured results of the hybrid system and CMM were calculated. Ten different panels were measured to assess the system accuracy, and for illustrative purposes, deviations of a featured hole in three vectors are shown in Figure 6.

From Figure 6, it can be seen that deviations between the measured values of the hybrid visual system and CMM look random and symmetric, but not uniformly distributed between minus and plus, which can be assumed to follow a normal distribution with the expected value of nonzero. According to the Lilliefors test for normality [22,23], we can confirm this normality assumption. Therefore, we can fit the experimental data with a normal distribution function, and the fitting results are shown in the right side of Figure 6. For all of the 58 featured points, most of the deviations between the measured results in these two systems are about 2–4 mm, and these errors are reproducible and consistently in the same direction. It can be safe to conclude that there are remarkable systematic errors in the measured results of this hybrid inspection system, which are mainly caused by the inaccuracy of the global calibration method.

To a large extent, the measuring accuracy of the hybrid inspection system has been limited by the robot positioning accuracy, as the industrial robots are normally designed for repeated work such as picking and placing, spot welding, and so on. The results showed high repeatability but low accuracy. For a KUKA KR16-2 (KUKA, Augsburg, Germany) industrial robot, its repeatability is up to ±0.05 mm, but its absolute accuracy can reach a few millimeters. Moreover, the discrepancy between the robot’s ideal and real behavior is due to the joint angle offsets, geometric link length inaccuracy, and non-geometric factors such as flexibility, backlash, thermal expansion, etc. As the robot normal model specified by the manufacturer has been used in the global calibration and measurement model of the hybrid system, the model inaccuracy will inevitably pass to the calibration and measurement results.

As the hybrid visual inspection system described in this paper is mainly applied for mass production such as automobile manufacturing, we can compensate the systematic error of the hybrid system based on the measured data of earlier products. That is, after the hybrid inspection system has been installed and debugged on-site, a few products (usually 10–20) are measured with the hybrid system and CMM, and an average error of each featured point is calculated as follows:

(24) $$\{\begin{array}{c} \Delta x=\sum_{i=1}^{n}(x_{CMM}-x_{hybrid})/n\\ \Delta y=\sum_{i=1}^{n}(y_{CMM}-y_{hybrid})/n\\ \Delta z=\sum_{i=1}^{n}(z_{CMM}-z_{hybrid})/n \end{array}$$

where *n* is the number of products measured for compensation. As the systematic errors are reproducible and consistent, we can use the average error above to represent it. An offset value is obtained for each featured point as offset=(·x,·y,·z). For the products measured during the later practical production process, the offset value is used to compensate the measured result as:

(25) $$\{\begin{array}{c} x_{comp}=x_{meas}+\Delta x\\ y_{comp}=y_{meas}+\Delta y\\ z_{comp}=z_{meas}+\Delta z \end{array}$$

where $\left(x_{meas},y_{meas},z_{meas}\right)$ is the measured result of the hybrid system, and $\left(x_{comp},y_{comp},z_{comp}\right)$ is the compensated result, which is the final result of the measurement system.

In order to verify the efficiency of the compensation method presented above and evaluate the final accuracy of the hybrid system, a follow-up study was performed on the compensated hybrid system. During the practical production process, hundreds of products were inspected on the hybrid station every day, and one of the products was picked out randomly and measured with CMM. The experiment lasted for one month and we obtained a dataset of 30 products. Deviations between measured results of the hybrid system and CMM were calculated.

Another way to evaluate the accuracy of the hybrid system is to calculate the correlation coefficient between the measured results of each featured point. The correlation coefficient determines the degree to which two variables are related. The range of the correlation coefficient is from −1 to 1. The value of 1 indicates that there is a perfect positive relationship between these two variables. For a positive increase in one variable, there is also a positive increase in another variable. The strength of the relationship varies in degree based on the value of the correlation coefficient. The correlation coefficient of *x* vector is calculated as follows:

(26) $$r=\frac{\sum_{i=1}^{n}\left(x_{i}-\bar{x}\right)\left(X_{i}-\bar{X}\right)}{\sqrt{\sum_{i=1}^{n}{\left(x_{i}-\bar{x}\right)}^{2}\cdot \sum_{i=1}^{n}{\left(X_{i}-\bar{X}\right)}^{2}}}=\frac{n\sum_{i=1}^{n}x_{i}X_{i}-\sum_{i=1}^{n}x_{i}\cdot \sum_{i=1}^{n}X_{i}}{\sqrt{n\sum_{i=1}^{n}{x_{i}}^{2}-{\left(\sum_{i=1}^{n}x_{i}\right)}^{2}}\cdot \sqrt{n\sum_{i=1}^{n}{X_{i}}^{2}-{\left(\sum_{i=1}^{n}X_{i}\right)}^{2}}}$$

where *x_i_* and *X_i_* are the measured values of the hybrid system and CMM. The deviation and correlation coefficient between the measured results of these two systems can be calculated on the three vectors (*x*, *y*, *z*) of each featured point.

For the 58 featured points on the rear underbody, there are 174 vectors, and the deviation and correlation coefficient were calculated for each vector. Statistics of the calculated results are shown in Table 1.

From Table 1, we can see that for the 174 measured vectors on the car rear underbody, deviations of all the vectors are less than ±0.5 mm, 96.55% vectors are less than ±0.3 mm, and correlation coefficient of 91.38% of vectors are larger than 0.6, while 85.64% vectors are larger than 0.7. Most of the dimension tolerances in the automobile manufacturing are set to be ±2 mm, a quality inspection system with an accuracy of ±0.5 mm is quite qualified for the job. So, it can be concluded that the compensated hybrid inspection system has a relatively high accuracy, which can be applied for the quality inspection in mass production such as automobile manufacturing.

## 5. Conclusions

A rapid global calibration technology for a hybrid visual inspection system has been presented in this paper. The hybrid system consists of an industrial robot with a flexible sensor and several stationary sensors, and the system global calibration has been divided into two steps, calibrating the flexible sensor first and then calibrating the stationary sensors. The flexible sensor global calibration method is performed based on the robot kinematic principle. With the calibrated flexible sensor, the stationary sensors are calibrated one by one based on the homography principle. Only a standard sphere and an auxiliary target with a 2D planar pattern are applied during the system global calibration; no sophisticated equipment is adopted, and it is convenient to re-perform during the hybrid system’s periodical maintenance. With an error compensation method, the final accuracy of the hybrid system is evaluated with the deviation and correlation coefficient between the measured results of the hybrid system and CMM. An accuracy verification experiment showed that the deviations of 96.55% of the featured points are below ±0.3 mm, and the correlation coefficients of 85.64% points are larger than 0.7. This has proved that the global calibration method is feasible and its accuracy is relatively high. Since it is easy and convenient to implement on-site, we expect that the proposed rapid global calibration technology will have wide application in the product quality of mass production. Future efforts will also be devoted to extend the application of hybrid visual inspection systems.

## Figures and Tables

**Table 1:** Statistics of the calculated deviation and correlation coefficients.

Deviation (mm)	Sum	Rate	Correlation Coefficient	Sum	Rate
[−0.1, 0.1]	37	21.26%	>0.9	28	16.09%
[−0.2, −0.1] & [0.1, 0.2]	82	47.13%	[0.8, 0.9]	58	33.33%
[−0.3, −0.2] & [0.2, 0.3]	49	28.16%	[0.7, 0.8]	63	36.21%
[−0.5, −0.3] & [0.3, 0.5]	6	3.45%	[0.6, 0.7]	10	5.74%
[<−0.5] & [>0.5]	0	0	[<0.6]	15	8.62%
